# Structure-Based Screening of Deep-Sea Microbial Metabolites Against *Plasmodium falciparum* Dihydroorotate Dehydrogenase

**DOI:** 10.3390/biology15050392

**Published:** 2026-02-27

**Authors:** Avtar Singh, Kannan R. R. Rengasamy, Soottawat Benjakul

**Affiliations:** 1 International Centre of Excellence in Seafood Science and Innovation, Faculty of Agro-Industry, Prince of Songkla University, Hat Yai 90110, Songkhla, Thailand; avtar.s@psu.ac.th; 2 Centre of Excellence for Pharmaceutical Sciences, North-West University, Potchefstroom 2520, South Africa; 3 Department of Food and Nutrition, Kyung Hee University, Seoul 02447, Republic of Korea

**Keywords:** deep-sea marine microbes, malaria, dihydroorotate dehydrogenase, dynamics simulation, ADME

## Abstract

Malaria is a major global health concern caused by *Plasmodium* parasites, among which *Plasmodium falciparum* is responsible for the most severe and fatal cases. The emergence of drug resistance to existing antimalarial therapies necessitates the discovery of novel molecular targets and chemically distinct inhibitors. Current study employed an integrated in silico drug discovery pipeline combining high-throughput structure-based virtual screening of 1549 deep-sea marine microbial metabolites with MM-GBSA binding free-energy estimation, QikProp-based ADME/Tox profiling, and 100 ns molecular dynamics (MD) simulations to link rapid screening with dynamic verification of binding stability. Molecular docking against *Plasmodium falciparum* dihydroorotate dehydrogenase (PfDHODH; PDB ID: 7KZ4) yielded five top-ranked compounds with Glide scores ranging from −12.02 to −10.61 kcal·mol^−1^, which is higher than the Primaquine (−6.920 kcal·mol^−1^; a clinically approved antimalarial reference compound). MM-GBSA analysis further refined hit selection, producing binding free energies (ΔG_bind) between −63.28 and −31.37 kcal·mol^−1^. The selected lead compounds included (±)-puniceusine P, aspergilol F, tersaphilone C, 4-carbglyceryl-3,3′-dihydroxy-5,5′-dimethyldiphenyl ether, and 15-O-methyl ML-236A. The top hits were subjected to 100 ns MD simulations in Desmond, demonstrating stable protein–ligand complexes, particularly for (±)-puniceusine P and 15-O-methyl ML-236A (protein backbone root mean square deviation (RMSD; ~0.8–1.0 Å). ADME profiling indicated acceptable predicted physicochemical and pharmacokinetic properties. Overall, these in silico findings highlight deep-sea marine microbial metabolites as promising PfDHODH inhibitor candidates requiring experimental validation.

## Simple Summary

Malaria remains a serious global health problem, partly because the parasite that causes the disease is becoming resistant to existing medicines. Thus, to address this challenge, the present study explores whether natural chemical substances from marine microorganisms could serve as starting points for new antimalarial drugs. These deep-sea organisms survive under extreme conditions and often produce unusual chemical structures that may interact with disease-causing proteins in new ways. Using advanced computer-based methods, we screened a large collection of deep-sea microbial compounds to identify those most likely to block a protein that the malaria parasite needs to survive. Several compounds showed strong and stable interactions with this protein and displayed properties consistent with drug-like behavior. Computer simulations further suggest that these compounds remain stably bound over time, supporting their potential effectiveness. Although laboratory testing is still required, our findings highlight deep-sea microbial compounds as promising candidates for future antimalarial drug development. This research may contribute to the long-term goal of discovering safer and more effective treatments for malaria.

## 1. Introduction

Malaria is one of the deadliest infectious diseases worldwide and is transmitted by female Anopheles mosquitoes carrying protozoan parasites of the genus Plasmodium. *Plasmodium falciparum*, *P. vivax*, *P. malariae*, *P. ovale*, and *P. knowlesi* are the five species of the genus Plasmodium that are generally known to cause malarial illnesses in humans. However, *P. falciparum* and *P. vivax* account for the majority of cases [1,2]. With an expected 241 million infections and 627,000 fatalities from malaria in 2020, it remains a significant worldwide health issue [1]. Despite decades of control efforts, malaria continues to impose a substantial disease burden, particularly in tropical and subtropical regions.

Malarial therapies have been extensively studied, and a wide range of antimalarial medications, including chloroquine and artemisinin, are now available for the prevention and treatment of malaria [2]. Artemisinin combination therapy (ACT) is the first-line treatment for *P. falciparum* malaria. ACT is the preferred first- and second-line treatment for uncomplicated *Plasmodium falciparum* malaria, as well as for *P. vivax* infections resistant to chloroquine. ACTs consist of a fast-acting artemisinin derivative, such as artesunate, artemether, or dihydroartemisinin, administered together with a longer-acting partner drug. The artemisinin component rapidly lowers parasite burden during the initial days of therapy, while the partner drug clears residual parasites, ensuring complete therapeutic cure [3]. However, antimalarials are among the most frequently prescribed and are somewhat affected by poor patient compliance, putting enormous pressure on *P. falciparum* parasites to develop resistance. The US Army’s high-throughput screening and drug discovery programs led to the development of several important antimalarial compounds, including pyrimethamine, the chloroquine analog amodiaquine, and the arylamino alcohol derivatives mefloquine and halofantrine. However, these agents also cause adverse effects and drug resistance [4,5]. Thus, the key limitation of existing antimalarial drug discovery is the relatively small number of clinically validated molecular targets and the structural similarity of many approved agents, which together increase selective pressure for cross-resistance. To sustain progress against malaria, drug discovery must therefore (i) identify and validate new *Plasmodium* targets that are essential to parasite survival, and (ii) discover novel chemical scaffolds that operate through distinct mechanisms of action and avoid existing resistance pathways.

Systems-level analytical strategies integrating transcriptomics, metabolomics, and pharmacological data have become powerful tools for elucidating how bioactive compounds modulate metabolic and signaling pathways beyond single-target effects, enabling mechanistic insight into complex biological responses [6]. Network pharmacology approaches that incorporate microbial and hepatic biotransformation further refine this framework by linking compound metabolism to therapeutic outcomes, particularly for natural products with multifaceted bioactivity [7]. Experimental studies on metabolic regulators such as astaxanthin demonstrate that small molecules can restore dysregulated metabolic pathways through enzyme- and pathway-level modulation, underscoring the relevance of metabolism-centered intervention strategies [8]. In parallel, real-world clinical evaluations of optimized antimicrobial regimens highlight the importance of mechanism-informed drug development to counter resistance and improve therapeutic efficacy in infectious diseases [9]. Comparative multicenter analyses further reinforce that rational, target-guided therapeutic strategies translate into measurable clinical benefits when grounded in mechanistic understanding [10]. Advances in genetic and quantitative trait analyses also emphasize how systematic identification of functional targets can guide intervention strategies in biologically complex systems [11].

In this context, *Plasmodium* dihydroorotate dehydrogenase (PfDHODH) has emerged as a validated and tractable target for antimalarial chemotherapy [12,13]. Even after target validation, the PfDHODH inhibitor space remains dominated by closely related chemotypes and faces documented resistance and pharmacokinetic challenges, including suboptimal oral bioavailability, metabolic instability, and exposure–response limitations. In general, PfDHODH catalyzes a rate-limiting step in de novo pyrimidine biosynthesis, and malaria parasites cannot salvage pyrimidines from the host [14]. Thus, PfDHODH is absolutely essential for parasite survival in the blood stage. Importantly, crystal structures reveal that the parasite DHODH is structurally distinct from the human dihydroorotate dehydrogenase (hDHODH). Key differences in the inhibitor-binding pocket allow selective inhibition. PfDHODH inhibitors such as DSM265 potently inhibit PfDHODH while sparing human hDHODH [15]. The availability of high-resolution PfDHODH structures (e.g., PfDHODH structures co-crystallized with DSM265) makes this protein highly amenable to in silico screening [16]. Despite strong validation of PfDHODH as a target, the inhibitor landscape remains dominated by closely related triazolopyrimidine scaffolds, which exhibit emerging on-target resistance and pharmacokinetic limitations, underscoring the need for structurally distinct chemotypes [17,18]. Moreover, many published PfDHODH series remain chemically related (triazolopyrimidines and close analogues) and exhibit pharmacokinetic or formulation challenges, so the discovery of structurally distinct scaffolds, especially from underexplored sources, remains an unmet need [19].

Natural products offer one promising route to novel chemotypes. The marine environment, in particular, is a rich source of unique bioactive molecules due to the extreme conditions (high pressure, low temperature, high salinity, etc.), leading to unusual chemistries and high structural diversity [20,21]. An overview of the broad classes of compounds (e.g., alkaloids, polyketides, terpenoids, etc.) and their microbial sources (such as fungi, bacteria, and other microorganisms) is provided in Table S1. These adaptive compounds often feature novel ring systems, halogenations, and stereochemistry that can translate into unique pharmacology. An antimalarial or antiparasitic active compound such as manzamine A, a complex alkaloid from a sponge-associated microbe, showed low-nanomolar inhibition of *P. falciparum* in vitro [22,23]. Similarly, a polyether metabolite isolated from *Streptomyces* sp. strain H668 exhibited antimalarial activity, highlighting the potential of marine-derived microbial secondary metabolites as antiparasitic agents [24]. Fattorusso and Taglialatela-Scafati [22] reported that about 60 secondary metabolites produced by marine organisms have been grouped into three structural types and discussed in terms of their reported antimalarial activities. Despite this chemical richness, relatively few marine metabolites, especially those from deep-sea microbial sources, have been systematically evaluated against PfDHODH [22,25]. Thus, given the complexity of structure and limited experimental accessibility of deep-sea metabolites, structure-based virtual screening (SBVS) is an efficient strategy to triage large or hard-to-access libraries such as curated deep-sea metabolite collections to a manageable set of chemically diverse candidates for experimental follow-up. Docking coupled with MM-GBSA rescoring helps prioritize ligands by predicted binding energy, while ADME filtering removes compounds with obvious drug-likeness liabilities before costly synthesis or biological testing. Finally, molecular dynamics provides an atomistic assessment of binding stability and interaction persistence that complements static docking poses. While MM-GBSA has known approximations (notably entropy and explicit water effects), it is widely used as a pragmatic, relative ranking tool in SBVS workflows [7,26].

This study aimed to identify novel inhibitors of *Plasmodium falciparum* dihydroorotate dehydrogenase (PfDHODH) by virtually screening a structurally diverse library of deep-sea marine metabolites. Using a structure-based approach, molecular docking against the PfDHODH active site, followed by MM-GBSA rescoring to estimate binding free energies, was performed. Top hits were further evaluated for pharmacokinetic properties through ADME prediction, and their dynamic behavior in complex with PfDHODH was assessed using molecular dynamics (MD) simulations. This integration in silico workflow is designed to discover chemically distinct and selective PfDHODH inhibitors that could serve as leads for next-generation antimalarial development. In this work, MarinLit curated library was used, which provides aggregated peer-reported marine natural products along with the detailed bibliographic provenance. Thus, this allows a focused screening of deep-sea metabolites with known isolation sources and structural assignments.

## 2. Experimental Section

### 2.1. Retrieval of Compound Structures

The 3D structures of 1549 compounds reported from deep-sea regions were extracted from the MarinLit (Royal Society of Chemistry, Cambridge, UK; https://marinlit.rsc.org/ (accessed on 16 March 2025), a dedicated database for marine natural products using the bibliography search option with the “deep-sea” filter, yielding 1549 compounds. The retrieved compounds were given as Supplementary Data (Supplementary Material S1).

### 2.2. Preparation of Target Protein

The 3D structures of the target proteins were prepared using Maestro module 13.7 (Maestro version, 2023-3) in the Schrodinger suite 2023-3. The crystal structure of *Plasmodium falciparum* dihydroorotate dehydrogenase (PfDHODH) bound to inhibitor DSM705 (**PDB: 7KZ4**) at a resolution of 1.75 Å was prepared using the Protein Preparation Wizard in the Maestro module with the OPLS4 force field. The FMN cofactor was retained during protein preparation and treated as an integral component of the PfDHODH active site throughout docking, MM-GBSA calculations, and MD simulations, thereby preserving the catalytically relevant protein environment. Protein protonation states were assigned and optimized using the Protein Preparation Wizard in Maestro, while ligand protonation and ionization states were generated using LigPrep with the Epik module at a physiological pH range of 7.0 ± 2.0 prior to docking.

The protein preparation process includes deleting water molecules; adding hydrogen atoms, side chains, and loops; and removing unwanted metals and ions. The ionization and tautomeric states of the heterogroups were adjusted. Finally, the optimization steps included optimizing the hydrogen bonds and refining the structure by limiting the root mean square deviation (RMSD to 0.3 Å). The active binding site of PfDHODH was defined based on the coordinates of the co-crystallized inhibitor DSM705, which is registered in the Protein Data Bank under the ligand code XC7 (PDB ID: 7KZ4). The co-crystallized ligand was subsequently removed prior to docking of the screened compounds.

### 2.3. Preparation of Ligand

Ligand preparation was carried out using the LigPrep module (Schrödinger Suite, Maestro version 13.7). Protonation and ionization states were generated using the Epik module at a target physiological pH of 7.0 ± 2.0. Relevant tautomeric states were also generated where applicable. All ligand structures were subsequently energy-minimized using the OPLS4 force field. During subsequent MD, up to 32 binding poses per ligand were generated by Glide to sample alternative conformations and orientations within the PfDHODH active site. Only unique, desalted parent structures were subjected to LigPrep processing to avoid redundancy during docking. The complete set of prepared ligand structures is provided in Supplementary Material S2.

### 2.4. Molecular Docking (MD) Process

MD was performed using the Glide module of the Schrödinger suite (Maestro version 13.7). Structure-based virtual screening was employed as an initial prioritization step because it enables efficient evaluation of large and chemically diverse compound libraries against a well-defined binding site while retaining atomic-level information on ligand–protein interactions [27]. This approach is particularly suitable for deep-sea natural product libraries, where experimental screening is constrained by limited compound availability and structural complexity.

The receptor grid was generated based on the PfDHODH active site defined by the co-crystallized inhibitor DSM705, which was removed prior to docking. Docking calculations were carried out using the extra-precision (XP) mode of Glide to enhance pose discrimination and reduce false-positive predictions. The prepared ligands were docked into the minimized PfDHODH structure, and the best-ranked poses were selected based on Glide XP scores in combination with favorable interaction patterns. The glide grid for 7KZ4 is provided in Supplementary Material S3.

Three-dimensional and two-dimensional protein–ligand interaction diagrams were analyzed using the Maestro workspace. Additionally, Primaquine was included as a clinically approved antimalarial reference compound for qualitative comparison of docking scores; it is noted that Primaquine is not a validated PfDHODH inhibitor. To validate the docking protocol, the co-crystallized reference ligand DSM705 was re-docked into the prepared PfDHODH structure, and the reproduced pose showed close agreement with the experimental binding conformation (Supplementary Material S4).

The docked compounds were ranked according to Glide XP scores, and approximately the top 10% of candidates were shortlisted for post-docking refinement, a pragmatic cutoff widely used in structure-based virtual screening to retain chemical diversity while maintaining computational feasibility [27]. These candidates were subsequently refined using MM-GBSA binding free-energy estimation, and only compounds with consistently favorable energetics were advanced to molecular dynamics simulations.

### 2.5. ADME/Tox

The QikProp module of Maestro Version 13.7 is an absorption, distribution, metabolism, and excretion (ADME) prediction tool that can generate certain descriptors of ADME. This module works based on ligand-based ADME/Tox prediction. The prepared ligands were used in fast mode. It predicts both pharmacologically and physicochemically significant descriptors. Based on Lipinski’s rule of five, ADME qualities evaluate the drug-like action of ligand molecules. Maestro Version 13.7 was used to analyze the ADME/T characteristics of the designed compounds [28].

### 2.6. Determination of Molecular Mechanics-Generalized Born Surface Area (MM-GBSA)

The MM-GBSA method is used to estimate the free energy of the screened ligands using representative binding poses obtained from molecular docking. MM-GBSA calculations were performed using the Prime module, where the binding free energy (ΔG_bind) was estimated from the molecular mechanics energy (ΔE_MM) and the solvation free energy (ΔG_solv) of the protein–ligand complex, receptor, and ligand.

The entropy term (−TΔS) was not explicitly calculated, as normal-mode entropy estimation is not included in the standard Prime MM-GBSA workflow. Therefore, the reported ΔG_bind values represent approximate relative binding energies suitable for comparative ranking rather than absolute free energies.

ΔGbind = ΔEMM + ΔGsolv − TΔS

Prior to MM-GBSA analysis, docked complexes were locally energy-minimized, and a single top-ranked pose per ligand was used for rescoring. Solvation effects were modeled using an implicit generalized Born solvation model.

Molecular docking analysis alone does not provide quantitative binding free energies or accurate affinity estimates for protein–ligand complexes; therefore, MM-GBSA rescoring was employed as a post-docking refinement step to improve hit prioritization and reduce false-positive docking results [29].

### 2.7. Determination of Ligand–Receptor Interaction (Simulations)

**Figure 1:**
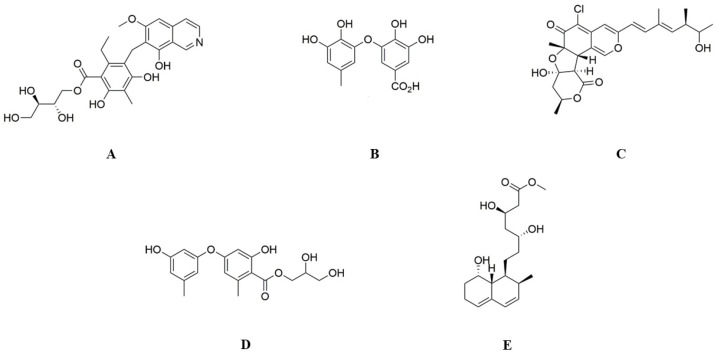
Chemical structures of the five top-ranked deep-sea marine microbial metabolites identified as predicted inhibitors of *Plasmodium falciparum* dihydroorotate dehydrogenase (PfDHODH) through structure-based virtual screening. Where: (**A**) (±)-puniceusine P, (**B**) aspergilol F, (**C**) tersaphilone C, (**D**) 4-carbglyceryl-3,3′-dihydroxy-5,5′-dimethyldiphenyl ether, and (**E**) 15-O-methyl ML-236A. Chemical structures were retrieved from the MarinLit database (Royal Society of Chemistry) and prepared using LigPrep (Schrödinger) prior to docking and molecular simulations.

The top-scoring compounds (Figure 1) were further evaluated for their stability in ligand–receptor interactions. The top-scoring compounds in the form of protein–ligand complexes were considered. The stability of the complex molecule was analyzed using MD simulation in the Schrödinger suite Desmond module (New York City, New York, USA). MD simulations assessed the dynamic stability and interaction persistence of the docked complexes rather than derived quantitative binding free energies. The prepared PfDHODH–ligand complexes were solvated in an explicit TIP3P water model within an orthorhombic simulation box, maintaining a 10 Å buffer distance from the protein surface. Counter ions and 0.15 M NaCl were added to neutralize the system and mimic physiological ionic strength. The systems were energy-minimized using the OPLS4 force field prior to simulation.

MD simulations were carried out for 100 ns under an NPT ensemble at a constant temperature of 300 K and a pressure of 1 atm, maintained using the Nosé–Hoover thermostat and the Martyna–Tobias–Klein barostat, respectively. A time step of 2 fs was used, and long-range electrostatics were treated using the particle mesh Ewald (PME) method.

All systems underwent an initial equilibration phase, and trajectory convergence was assessed prior to production analysis. Although only a single MD trajectory was generated for each complex, this approach is widely used for exploratory stability assessment in structure-based virtual screening workflows.

The simulation trajectories were analyzed to determine protein backbone RMSD, root mean square fluctuation (RMSF), radius of gyration (Rg), protein–ligand hydrogen bonding patterns, and overall potential energy stability. Protein secondary structure elements and interaction persistence were also monitored throughout the simulation period. All MD simulation data and analyses are provided in Supplementary Material S5.

Compounds were evaluated using docking scores, MM-GBSA binding energies, ADME descriptors, and MD stability metrics.

## 3. Results

### 3.1. Docking Results and Binding Mode Analysis

The prepared ligands were checked for any duplicate entries, and the output of ligprep file is provided in the Supplementary Files S2. The objective of ensuring the precise attribution of formal charges and applying force field treatment was accomplished by including any missing hydrogen atoms and appropriately determining the ionization states [30].

### 3.2. Molecular Modelling Studies

MD has gained significant prominence in drug discovery, which enables modelling of atomic-level interactions between a protein and a ligand. This approach facilitates the characterization of ligand behavior within the binding site of target proteins and the elucidation of fundamental biochemical processes [31]. Following the setup of the grid box for PfDHODH using the Receptor Grid Generation tool of Glide in Maestro, the prepared 3D molecular structures were subjected to docking within the binding site of PfDHODH, where the inhibitor was co-crystallized.

Table 1 depicts the outcomes of the five selected docked ligands, with the highest negative docking scores falling within the range of −12.02 to −10.61 kcal/mol. It should be emphasized that docking scores provide a relative ranking rather than an absolute binding affinity. Hence, the current study prioritized compounds based on a consensus workflow (top Glide XP scores followed by MM-GBSA rescoring and visual inspection of binding poses) rather than an arbitrary fixed score cutoff. Importantly, these docking scores are comparable to or exceed those of the co-crystallized PfDHODH reference inhibitor (DSM265), indicating that the identified marine microbial metabolites exhibit binding affinities within a range considered promising for PfDHODH inhibition in structure-based screening studies. For comparison, the clinically approved antimalarial primaquine yielded a significantly weaker docking score (−6.920 kcal/mol).

**Figure 2:**
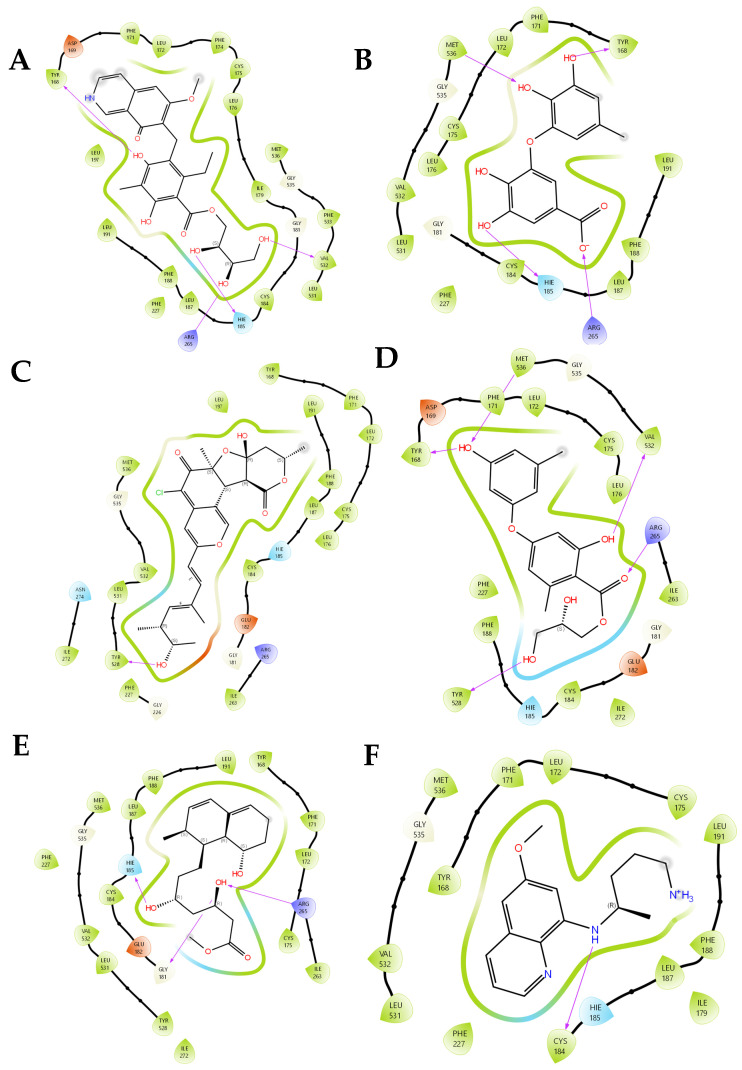
Two-dimensional protein–ligand interaction diagrams for the five selected deep-sea marine microbial metabolites bound to PfDHODH: (**A**) (±)-puniceusine P, (**B**) aspergilol F, (**C**) tersaphilone C, (**D**) 4-carbglyceryl-3,3′-dihydroxy-5,5′-dimethyldiphenyl ether, (**E**) 15-O-methyl ML-236A, and (**F**) Co-crystal ligand. Interaction types are color-coded according to the default Desmond classification scheme (hydrogen bonds: green dashed lines; hydrophobic contacts: pink arcs; π-π stacking: orange lines; ionic interactions: red dashed lines; water-mediated contacts: blue lines). Primaquine was included as a clinically approved antimalarial reference compound for qualitative comparison; it is not a validated PfDHODH inhibitor.

In the current study, no single numeric cutoff was used to define a ‘hit’; instead, top-scoring compounds were advanced when they met multiple criteria, including favorable Glide XP scores along with consistent MM-GBSA ΔG bind estimates and reasonable QikProp properties. In addition, plausible binding poses occupying key subpockets were considered because small differences in Glide score alone are not necessarily statistically meaningful in isolation. The process through which the ligands interact with the target is illustrated in Figure 2. Hydrogen bond connections are thought to play a pivotal role in the interactions between proteins and ligands. The ability to make accurate predictions regarding the affinity between proteins and ligands has played a crucial role in driving advancements in drug discovery in the pharmaceutical industry.

### 3.3. Molecular Mechanics-Generalized Born Surface Area

MM-GBSA calculations were performed to further improve the ligand ranking and determine the displayed binding energies. The calculated values yielded approximate binding free energies, with a more negative value corresponding to a higher binding intensity. Table 2 also illustrates the binding energies of the studied ligands within the binding site of PfDHODH. The binding free energy (ΔG bind) values for the targeted protein and each ligand were computed using the MMGBSA method and are presented in Table 1. 15-O-methyl ML-236A exhibited the highest binding free change in energy (ΔG bind), whereas tersaphilone C demonstrated the lowest ΔG binding [29]. MM-GBSA was therefore used as a rescoring step to refine docking-derived rankings rather than as an absolute predictor of affinity. In practice, compounds were deprioritized only when MM-GBSA results contradicted favorable docking poses and visual inspection revealed poor complementarity or solvent-exposed polar groups that would reduce effective binding. Thus, MM-GBSA rescoring further strengthened hit prioritization by refining docking poses and highlighting compounds with consistently favorable binding energetics, with ΔG_bind values comparable to those reported for known PfDHODH inhibitors in previous computational studies.

We observed instances where favorable docking scores did not translate into top MM-GBSA rankings. Such discrepancies can arise because docking scoring functions emphasize geometric complementarity and local interactions, whereas MM-GBSA additionally accounts for molecular mechanics energies and solvation effects; for example, compounds that present favorable shape complementarity but expose polar groups to bulk solvent can receive worse MM-GBSA rescoring. Where such discrepancies occurred in our set, we examined the poses for solvent-exposed polar groups, unfavorable strain, or lack of key conserved interactions, and deprioritized the compound if these issues persisted.

### 3.4. In Silico Pharmacokinetic Profile

Assessment of pharmacokinetic properties is essential to prioritize computational hits with acceptable drug-like characteristics before experimental validation. Therefore, predicted ADME descriptors are routinely used to assess whether virtual screening hits possess properties compatible with oral bioavailability and systemic exposure. The predicted ADME descriptors were used to evaluate whether the top-ranked PfDHODH inhibitors fall within ranges compatible with oral bioavailability and systemic exposure. In silico pharmacokinetic profiles (absorption, distribution, metabolism, and excretion), which have been predicted using QikProp are shown in Table 3. Pharmacokinetic prediction was employed to evaluate the physicochemical properties, biological activities, and drug-like characteristics of a molecule. These descriptors are widely applied to flag potential absorption or permeability liabilities prior to experimental testing.

The selected deep-sea marine metabolites were subjected to prediction of various descriptors, including molecular weight, number of rotatable bonds, number of heavy atoms, number of hydrogen bond donor and acceptor traits, octanol/water coefficient, aqueous solubility, brain/blood partition coefficient, human oral absorption, and bioavailability of the selected compounds. Together, these parameters provide insight into solubility-driven absorption, membrane permeability, and overall drug-likeness. In comparison to all other compounds, (±)-puniceusine P and tersaphilone C had the largest molecular masses of 487.505 and 476.953 g/mol, respectively. These molecules also have the largest solvent-accessible surface areas and are the most hydrophobic components. Compared to the other compounds and their acceptable range, the polar surface areas of compounds (±)-puniceusine P and aspergilol F were slightly higher. All compounds fell within the range of hydrogen bond donors and acceptors. Water/gas (QPlogPw), aqueous solubility (QPlogS), brain/blood (QPlogBB), and skin permeability (QPlogKp) were among the five predicted partition coefficients that fell within the recommended ranges, whereas octanol/gas (QPlogPoct) was above and octanol/water (QPlogPo/w) was below. These trends indicate differences in lipophilicity that may influence oral absorption efficiency.

The greatest predicted water solubility of −2.476 (QPlogS) for aspergilol F, when compared to all the other compounds, which are within the range of −6.144 to −2.476, is significant even if all ligands are categorized as poor in human oral absorption (HOA). Because low solubility restricts absorption and results in low oral bioavailability, a medication with high oral bioavailability may result in a reduction in the dosage required to produce the desired pharmacological effect. These prediction results show that tersaphilone C, which has a human oral absorption of 93.88% compared with the other four compounds, may be a more effective drug candidate in terms of its efficacy. However, the three partition values for octanol/gas, water/gas, and octanol/water imply that aspergilol F has low lipophilicity, which may result in poor rates of passive diffusion and gastrointestinal absorption. The ability of a medicinal candidate to penetrate the brain is another crucial factor. Tersaphilone C had the smallest anticipated brain/blood partition coefficient (QPlogBB) of all ligands, but it was still within the recommended range of −1.337. This suggests limited central nervous system exposure while remaining within acceptable pharmacokinetic limits.

The unfavorable characteristics of aspergilol F and (±)-puniceusine P can be attributed to their slightly larger polar surface area. To improve its lipophilicity and oral absorption, the structures of (±)-puniceusine P and aspergilol F may be further optimized by removing a specific polar surface area. Accordingly, structural optimization aimed at reducing excess polarity may improve lipophilicity and oral absorption in future lead optimization efforts [32].

### 3.5. Simulation of Ligand–Receptor Interaction

An integrated comparison of docking performance, MM-GBSA binding energies, ADME descriptors, and molecular dynamics stability metrics for the top-ranked compounds is summarized in Table 4. Molecular dynamics simulation was performed to determine the stability, conformation, and intermolecular interactions of the ligand molecules with the target protein. Using the Desmond package, time-dependent changes in the complexes were calculated over 100 ns. The MD simulation was performed at the specified volume, density, pressure, and temperature conditions dictated by thermodynamics. Using the ensembles, the entire system was annealed and reached equilibrium. Additionally, the final step of the procedure examined the structural changes of the complex.

**Figure 3:**
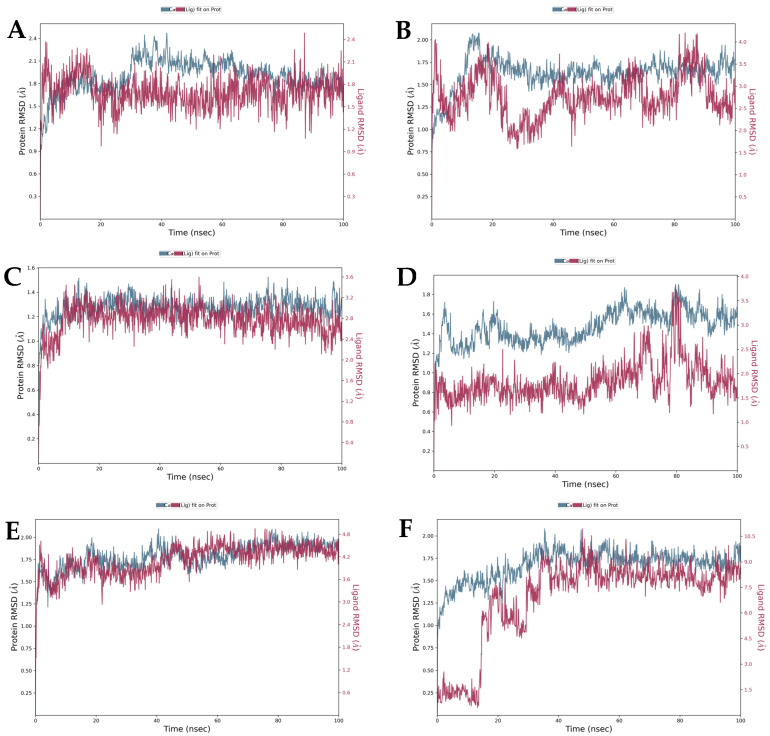
Root mean square deviation (RMSD) plots of PfDHODH backbone (blue) and ligand heavy atoms (red) during 100 ns molecular dynamics simulations for complexes with (**A**) (±)-puniceusine P, (**B**) aspergilol F, (**C**) tersaphilone C, (**D**) 4-carbglyceryl-3,3′-dihydroxy-5,5′-dimethyldiphenyl ether, (**E**) 15-O-methyl ML-236A, and (**F**) Co-crystal ligand. Primaquine was included as a clinically approved antimalarial reference compound for qualitative comparison; it is not a validated PfDHODH inhibitor.

**Figure 4:**
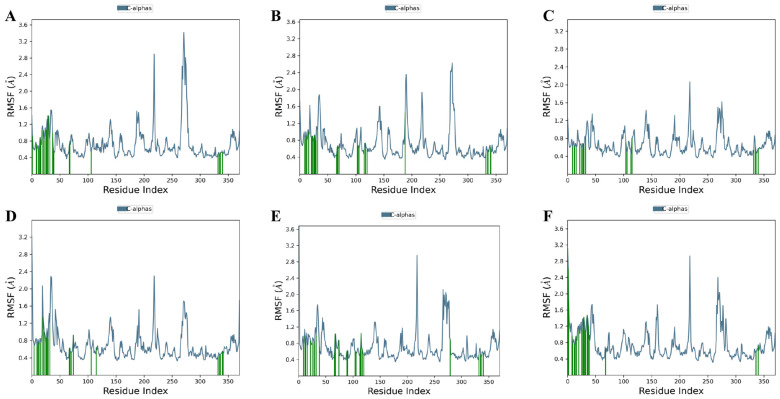
Root mean square fluctuation (RMSF) profiles of PfDHODH residues during 100 ns molecular dynamics simulations in complex with (**A**) (±)-puniceusine P, (**B**) aspergilol F, (**C**) tersaphilone C, (**D**) 4-carbglyceryl-3,3′-dihydroxy-5,5′-dimethyldiphenyl ether, (**E**) 15-O-methyl ML-236A, and (**F**) Co-crystal ligand. Primaquine was included as a clinically approved antimalarial reference compound for qualitative comparison; it is not a validated PfDHODH inhibitor.

**Figure 5:**
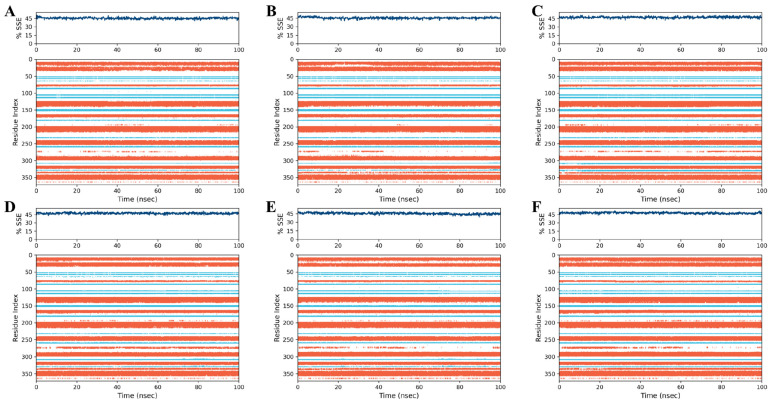
Protein secondary structure element (SSE) timeline of PfDHODH during 100 ns molecular dynamics simulations for complexes with (**A**) (±)-puniceusine P, (**B**) aspergilol F, (**C**) tersaphilone C, (**D**) 4-carbglyceryl-3,3′-dihydroxy-5,5′-dimethyldiphenyl ether, (**E**) 15-O-methyl ML-236A, and (**F**) Co-Crystal ligand. Primaquine was included as a clinically approved antimalarial reference compound for qualitative comparison; it is not a validated PfDHODH inhibitor.

To assess the degree of structural changes, the trajectories of each complex were subjected to several specific parameters, including RMSD, RMSF, protein secondary structure elements (SSEs), conformational modification of ligands, and intermolecular interactions. The results indicate that four of the five tested marine metabolites—(±)-puniceusine P, aspergilol F, tersaphilone C, and 15-O-methyl ML-236A—formed stable ternary complexes with PfDHODH over the full 100 ns simulation. In contrast, 4-carbglyceryl-3,3′-dihydroxy-5,5′-dimethyldiphenyl ether exhibited persistent structural instability, characterized by large RMSD fluctuations and failure to converge (Figure 3, Figure 4 and Figure 5). Convergence and equilibration behavior were assessed by monitoring the plateau of backbone RMSD and the stability of system potential energy. In our trajectories, backbone RMSD reached a stable plateau by ~20 ns, and total potential energy fluctuations were small thereafter, indicating reasonable equilibration for the 100 ns production runs. According to equilibration, the complex structures of all four compounds varied slightly (0.8–1 Å). Aspergilol F exhibits a small fluctuation at 80 nanoseconds, and 4-carbglyceryl-3,3′-dihydroxy-5,5′-dimethyldiphenyl ether exhibits significantly fluctuating behavior. Per-residue RMSF analysis indicates that residues lining the catalytic pocket (including TYR168, HIE185, ARG265, TYR528 and VAL532) generally display low fluctuation in complexes with the most stable ligands, supporting persistent ligand contacts during the MD trajectories (Figure 4). The system stabilized at approximately 20 ns of equilibration, and the stability of the compound was maintained up to 100 ns. The protein–ligand intermolecular interactions throughout the MD simulations support the binding potency and stability of deep-sea marine metabolites with the active site amino acids.

To better evaluate interaction persistence, the time-resolved occupancy of key hydrogen bonds and hydrophobic contacts over the 100 ns trajectories was also analyzed. For the most stable complexes ((±)-puniceusine P and 15-O-methyl ML-236A), several hydrogen bonds with catalytic residues (TYR168, HIE185, and ARG265) were observed to persist for the majority of the simulation window, whereas in less stable complexes, these contacts were intermittent or absent. Comparative MD behavior versus the co-crystallized reference ligand showed that the top hits maintained similar or improved backbone RMSD stability and comparable persistence of key interactions, supporting the notion that these marine metabolites engage the PfDHODH active site in a mechanistically relevant manner.

## 4. Discussion

Given the ongoing global burden and the emergence of drug resistance, discovery of novel antimalarial scaffolds and validated molecular targets remains an urgent priority. PfDHODH is a compelling antimalarial target because it catalyzes an essential parasite-specific step in de novo pyrimidine biosynthesis and because the parasite cannot recover host pyrimidines. Thus, selective inhibition causes parasite death while allowing a large therapeutic window. Prior DHODH inhibitors (e.g., DSM265) validate the target clinically but have also revealed that on-target resistance can emerge from point mutations in the DHODH binding pocket, a problem that can be mitigated by discovering chemically diverse scaffolds that bind differently to the active site [33].

A wide variety of natural marine products with unique chemical structures and bioactivities can be found in the deep-sea. Deep-sea microbial metabolites often contain unusual ring systems, halogenations, and stereochemistry that can confer distinct interactions with protein targets, making them attractive sources for novel chemotypes. In this study, we evaluated deep-sea microbial metabolites using structure-based drug discovery and virtual screening [34].

Five compounds were selected from a chemical library based on their structural diversity using structure-based drug design. The molecular interactions between the co-crystallized pyrrole-based dihydroorotate dehydrogenase inhibitors (DSM705) and the binding site of 7KZ4 were identified by analysis. By examining the binding conformation of the ligands, it was also possible to determine the atomic-level molecular recognition of the targeted *Plasmodium falciparum*–ligand complex.

Overall, the combined docking, MM-GBSA, and MD results indicate that several deep-sea microbial metabolites can stably engage the PfDHODH active site, supporting their prioritization for further evaluation. To clarify the molecular interactions of 7KZ4 with docked ligands, all ligands were investigated based on the docking score and predicted binding energy [35]. Tersaphilone C displayed the forecasted lowest binding energy of −31.37 kcal/mol in the binding site of 7KZ4, whereas 15-O-methyl ML-236A displayed the highest negative binding energy of around −63.28 kcal/mol.

(±)-Puniceusine P forms hydrogen bonds with the binding site residues of 7KZ4. The PfDHODH binding site residues TYR168, HIE185, and VAL532, with bond lengths of 2.10, 2.09, and 1.96 Å, displayed hydrogen bonding with the different hydroxyl groups of (±)-puniceusine P. Aspergilol F demonstrated hydrogen bond interactions with residues TYR168 (1.94 Å), HIE185 (2.11 Å), while forming a long-range electrostatic contact with ARG265 (4.91 Å), a residue critical for substrate binding and catalytic loop stabilization.

Interactions between tersaphilone C and the PfDHODH binding site were made through single residues similar to those of the DHODH inhibitor, that is, hydrogen bonds with residue TYR528, with a bond length of 1.94 Å. 4-Carbglyceryl-3,3′-dihydroxy-5,5′-dimethyldiphenyl ether displayed interactions with residues that were similar to TYR168 and ARG265 via hydrogen bonds, with bond lengths of 1.86 and 1.95 Å, as well as with other residues like MET536, VAL532, and TYR528 via hydrogen bonds, with bond lengths of 2.65, 2.02, and 1.80 Å.

15-O-methyl ML-236A shares key interactions with (±)-puniceusine P and aspergilol F, a notably HIE185 (2.23 Å) and ARG265 (2.07 Å), while uniquely engaging GLY181 (2.01 Å), a residue located in the flexible hinge region that modulates active-site accessibility. Unlike the other compounds, 15-O-methyl ML-236A fully occupies the substrate-binding pocket with high shape complementarity, mirroring the binding mode of the co-crystallized inhibitor DSM705 (PDB: 7KZ4).

These interaction patterns provide a structural rationale for the stability trends observed in subsequent molecular dynamics analyses. Where available, the docking and MM-GBSA scores of the top hits were compared to the co-crystallized reference inhibitor (DSM705) to provide context for ranking (Table 1 and Table 2). Although some hits approach or match the reference in docking/MM-GBSA ranking, direct biochemical comparisons are needed because docking/MM-GBSA are relative predictors. Therefore, experimental DSM265/DSM705 IC_50_ benchmarks remain the definitive comparator.

The limited interaction profile of Tersaphilone C (single TYR528 contact) explains its substantially weaker binding affinity (−31.37 kcal/mol) compared to compounds engaging the catalytic triad. We conclude that these ligands represent computationally prioritized candidates with potential antimalarial relevance based on their docking scores, hydrogen bonds, binding energies, and physicochemical and pharmacokinetic features. A molecular dynamics (MD) simulation was performed to determine the stability of the ligand molecules and how they interacted with the targeted protein [30]. The Desmond package was used, and the time-dependent changes in the complexes were run for over 100 ns.

The findings showed that, with the exception of compound 4 (BMCL12), which was unstable during the specified time period, all the compounds could form a stable ternary complex with the targeted protein 7KZ4. All complexes equilibrated by ~20 ns, and with the exception of 4-carbglyceryl-3,3′-dihydroxy-5,5′-dimethyldiphenyl ether, backbone RMSD remained around ~0.8–1.0 Å for the remainder of the trajectories (Figure 3). An RMSD of ~0.8–1.0 Å for the protein backbone indicates that the protein–ligand complex maintained its overall fold and that the ligand did not induce large conformational rearrangements; pharmacologically, this supports sustained occupation of the binding pocket under simulated conditions and therefore increases the plausibility of a stable inhibitory complex. However, RMSD stability is a structural indicator rather than a direct measure of biochemical potency; enzymatic inhibition (IC_50_) and cellular activity remain necessary to translate structural stability into pharmacological effect [36].

Additionally, the flexibility of the complexes was examined along with the ligands found in the active site of the protein. The variation in the complexes as a function of time was examined by RMSF The N-terminus fluctuated more than the C-terminus (RMSF values ~2.5 Å vs. ~2.2 Å). Residue-level RMSF analysis shows that catalytic pocket residues (TYR168, HIE185, ARG265, TYR528, VAL532) generally exhibit low fluctuation in the most stable complexes, supporting the hypothesis that these ligands form persistent contacts with catalytically relevant residues (Figure 4). RMSF traces indicate that, aside from the unstable compound noted above, the catalytic region exhibited low per-residue fluctuation, consistent with stable ligand engagement (Figure 4). With the exception of 4-carbglyceryl-3,3′-dihydroxy-5,5′-dimethyldiphenyl ether, which had poor intermolecular contact with the protein, the marine metabolite complexes were very stable in the catalytic area (Figure 5).

**Figure 6:**
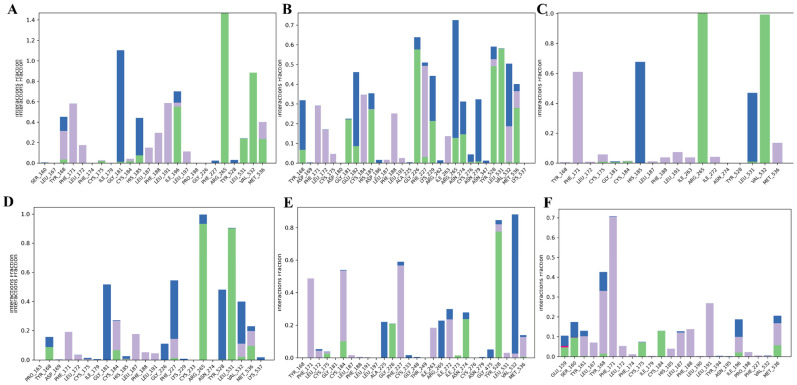
Protein–ligand interaction histograms summarizing hydrogen bonds, hydrophobic contacts, and other intermolecular interactions observed during 100 ns molecular dynamics simulations for PfDHODH complexes with (**A**) (±)-puniceusine P, (**B**) aspergilol F, (**C**) tersaphilone C, (**D**) 4-carbglyceryl-3,3′-dihydroxy-5,5′-dimethyldiphenyl ether, (**E**) 15-O-methyl ML-236A, and (**F**) Co-Crystal ligand Interaction types are color-coded according to the default Desmond classification scheme, including hydrogen bonds, hydrophobic contacts, ionic interactions, and water-mediated bridges.

To further characterize the stability and persistence of intermolecular contacts, protein–ligand interaction histograms were generated over the 100 ns simulations. These histograms summarize hydrogen bonding, hydrophobic interactions, and other non-covalent contacts formed between PfDHODH and each ligand throughout the simulation (Figure 6). During the simulation, SSE (alpha helices and beta strands) were observed. To anticipate the binding mode of each of the five molecules in the binding region of the targeted protein, an atomic-level understanding is crucial. Intermolecular interactions including hydrogen bonds, hydrophobic contacts, and ionic interactions were analyzed throughout the 100 ns MD simulations to assess binding mode stability and persistence. Importantly, the time-resolved interaction analysis indicates that many key hydrogen bonds and hydrophobic contacts are conserved relative to known PfDHODH inhibitor interaction patterns, and several interactions involve catalytically or structurally important residues, suggesting on-target inhibition consistent with disruption of pyrimidine biosynthesis, a mechanism validated by clinical DHODH inhibitors like DSM265.

Several leads present chemotypes (rigid polycyclic cores, halogenations, and distinct hydrogen-bonding geometries) that are chemically different from the triazolopyrimidine series that dominates reported PfDHODH inhibitors; these features could enable alternative subpocket engagement or halogen–π interactions and may help retain activity against DHODH variants selected by classical chemotypes [37]. However, experimental cross-resistance profiling will be required to validate this hypothesis. However, these computational observations are predictive and require biochemical PfDHODH assays, cellular antiplasmodial testing, and expanded ADME/toxicity evaluation to confirm antimalarial activity and drug-like suitability. Experimental cross-resistance profiling will be required to validate this hypothesis.

Overall, the five lead compounds identified in this study belong to chemical classes that are well documented for their biological relevance in marine-derived natural products. Marine alkaloids, polyketides, and related secondary metabolites produced by marine microorganisms have been widely reported to exhibit antiparasitic and enzyme inhibitory activities, including antimalarial effects [24,25]. Several marine microbial metabolites have been shown to interfere with mitochondrial function and essential metabolic pathways required for Plasmodium survival, including pyrimidine biosynthesis, in which PfDHODH plays a critical role [8,9,24]. In this context, the stable binding of the selected compounds within the PfDHODH active site and the persistence of key protein–ligand interactions observed during molecular dynamics simulations provide a plausible mechanistic basis for their potential antimalarial relevance. Although direct experimental evidence of PfDHODH inhibition by these molecules is beyond the scope of the present study, the combination of favorable binding energetics, interaction stability, and literature-reported biological activities of structurally related marine microbial metabolites supports their prioritization for future experimental validation.

## 5. Conclusions

The present study demonstrates that computational approaches can prioritize candidate inhibitors of an essential malarial enzyme, supporting early-stage antimalarial drug discovery. For further validation, in vitro investigations are required. A chemical library of natural deep-sea marine compounds was used in this study, and five compounds were selected based on docking scores and binding energies. It is projected that (±)-puniceusine P and 15-O-methyl ML-236A could be inhibitors of *P. falciparum*. Tersaphilone C (Ste13) should be derivatized to enhance its ADME profile. The data presented here can be useful in identifying lead molecules for future in vitro and in vivo studies, as well as for drug discovery and development against malaria because, although there are currently many treatment remedies for the disease, the need for novel drugs is increasing due to the rising infection rate. Furthermore, future studies should be focused on comprehensive in silico and experimental toxicity assessments to further evaluate the safety profile of the identified lead compounds.

## Figures and Tables

**Table 1:** Docking validation of top-scoring compounds.

Compound Name	Docking Score (kcal/mol)	Glide g Score (kcal/mol)	Glide Energy	Interacting Residues	Bond Length (Å)
(±)-puniceusine P	−11.990	−12.022	−59.458	TYR168	2.10
				HIE185	2.09
				VAL532	1.96
Aspergilol F	−11.504	−11.512	−44.3046	TYR168	1.94
				HIE185	2.11
				ARG265	4.91
Tersaphilone C	−10.950	−10.954	−47.2558	TYR528	1.94
4-carbglyceryl-3,3′-dihydroxy-5,5′-dimethyldiphenyl ether	−10.819	−10.830	−54.0097	TYR168	1.86
				MET536	2.65
				VAL532	2.02
				TYR528	1.80
				ARG265	1.95
15-O-methyl ML-236A	−10.611	−10.611	−48.7999	HIE185	2.23
				GLY181	2.01
				ARG265	2.07
* Primaquine	−6.920	−6.920	−41.201	CYS184	2.08

* Primaquine was included as a clinically approved antimalarial reference compound for qualitative comparison; it is not a validated PfDHODH inhibitor.

**Table 2:** MM-GBSA results of top-scoring compounds.

Compound Name	MM-GBSA Binding Energy (kcal/mol)
(±)-puniceusine P	−55.14
Aspergilol F	−36.10
Tersaphilone C	−31.37
4-carbglyceryl-3,3′-dihydroxy-5,5′-dimethyldiphenyl ether	−54.03
15-O-methyl ML-236A	−63.28
* Primaquine	−47.33

* Primaquine was included as a clinically approved antimalarial reference compound for qualitative comparison; it is not a validated PfDHODH inhibitor.

**Table 3:** ADME Tox prediction of the top-scoring deep-sea marine compounds.

Lead Molecule	Physiochemical Parameters					Pharmacokinetic Parameters			Drug Likeness Properties	
	Rotatable Bonds	Hydrogen Acceptor	Hydrogen Donor	Heavy Atoms	MW (Da)	Mop	GI Absorption	BBB Permeation	Lipinski Rule	Bioavailability
(±)-puniceusine P	14	1	1	15	487.505	3.87	High	Yes	0	0.55
Aspergilol F	16	2	0	20	292.245	4.67	High	No	0	0.55
Tersaphilone C	13	0	0	16	476.953	6.44	Low	No	1	0.55
4-carbglyceryl-3,3′-dihydroxy-5,5′-dimethyldiphenyl ether	1	5	3	20	348.352	0.52	High	No	0	0.55
15-O-methyl ML-236A	3	6	2	23	338.443	0.47	High	No	0	0.55

**Table 4:** Integrated summary of docking, MM-GBSA, ADME, and molecular dynamics stability metrics for top PfDHODH ligands.

Compound	Glide XP Score (kcal·mol^−1^)	MM-GBSA ΔG_bind (kcal·mol^−1^)	MD Stability (RMSD, Å)	Key Interactions	ADME Highlight	Overall Priority
(±)-Puniceusine P	−12.022	−55.14	~0.8–1.0 (stable)	TYR168, HIE185, VAL532	Moderate HOA; acceptable solubility	High
Aspergilol F	−11.512	−36.10	~0.9–1.0 (stable)	TYR168 HIE185 ARG265	High aqueous solubility (QPlogS)	Moderate–High
Tersaphilone C	−10.954	−31.37	~1.2 (stable)	TYR528	Highest HOA (~93.9%)	Moderate
4-Carbglyceryl-3,3′-dihydroxy-5,5′-dimethyldiphenyl ether	−10.830	−54.03	~1.0–1.6 (unstable)	TYR168 VAL532 ARG265 TYR528 MET536	Poor oral absorption	Low
15-O-Methyl ML-236A	−10.611	−63.28	~1.0 (stable)	HIE185, ARG265, GLY181	Drug-like MW; acceptable ADME	High

## Data Availability

Data will be available on request.

## Supplementary Materials

The following supporting information can be downloaded at: https://www.mdpi.com/article/10.3390/biology15050392/s1. All supporting data related to compound selection, ligand preparation, docking, MM-GBSA analysis, and molecular dynamics simulations are provided as Supplementary Materials. These include the deep-sea compound library (Supplementary Material S1), prepared ligand files (Supplementary Material S2), receptor grid files (Supplementary Material S3), docking validation data (Supplementary Material S4), and molecular dynamics trajectories and analyses (Supplementary Material S5), ensuring transparency and reproducibility of the computational workflow. An overview of the chemical classes and microbial origins of the screened compounds is provided in Table S1. References [38,39,40,41] are cited in the supplementary materials (Table S1).
