# Supplementary material for: Structure-Based Screening of Deep-Sea Microbial Metabolites Against Plasmodium falciparum Dihydroorotate Dehydrogenase
Source: Biology (Basel). 2026 Feb 27;15(5):392. doi: 10.3390/biology15050392 (PMC12984942; doi:10.3390/biology15050392)
Supplement: Supplementary file 1 [file biology-15-00392-s001.zip › biology-4056089-supplementary/Supp Files/Table S1.pdf]

Table S1. An overview of the chemical classes and microbial origins of the screened compounds.

**Compound class**	**Typical microbial sources**	**Representative references**
Alkaloids	Marine-derived fungi (*Aspergillus*, *Penicillium*), actinomycetes (*Streptomyces* spp.)	Blunt et al., 2018; Carroll et al., 2020
Polyketides	Marine bacteria and fungi, especially *Streptomyces* and filamentous fungi	Newman & Cragg, 2020; Carroll et al., 2020
Terpenoids	Marine-derived fungi and bacteria	Mayer et al., 2019; Blunt et al., 2018
Peptides / Depsipeptides	Marine bacteria, cyanobacteria, actinomycetes	Mayer et al., 2019; Carroll et al., 2020
Lactones	Marine fungi and bacteria	Blunt et al., 2018
Hybrid PKS–NRPS metabolites	Marine actinomycetes and fungi	Newman & Cragg, 2020

References.

[1] Blunt, J.W.; Carroll, A.R.; Copp, B.R.; Davis, R.A.; Keyzers, R.A.; Prinsep, M.R. Marine natural products. *Nat. Prod. Rep.* **2018**, *35*, 8–53. https://doi.org/10.1039/C7NP00052A

[2] Carroll, A.R.; Copp, B.R.; Davis, R.A.; Keyzers, R.A.; Prinsep, M.R. Marine natural products. *Nat. Prod. Rep.* **2020**, *37*, 175–223. https://doi.org/10.1039/C9NP00069K

[3] Mayer, A.M.S.; Guerrero, A.J.; Rodríguez, A.D.; Taglialatela-Scafati, O.; Nakamura, F.; Fusetani, N. Marine pharmacology in 2014–2016. *Mar. Drugs* **2019**, *17*, 364. https://doi.org/10.3390/md17080364

[4] Newman, D.J.; Cragg, G.M. Natural products as sources of new drugs. *J. Nat. Prod.* **2020**, *83*, 770–803. https://doi.org/10.1021/acs.jnatprod.9b01285
